# Physical, social, psychological and existential trajectories of loss and adaptation towards the end of life for older people living with frailty: a serial interview study

**DOI:** 10.1186/s12877-016-0350-y

**Published:** 2016-10-20

**Authors:** Anna Lloyd, Marilyn Kendall, John M. Starr, Scott A. Murray

**Affiliations:** 1 Primary Palliative Care Research Group, The Usher Institute of Population Health Sciences & Informatics, The University of Edinburgh, Medical School, Teviot Place, Edinburgh, EH8 9AG UK; 2 Centre for Education and Research, St Columba’s Hospice, 17 Boswall Road, Edinburgh, EH5 3RW UK; 3 Alzheimer Scotland Dementia Research Centre, The University of Edinburgh, 7 George Square, Edinburgh, EH8 9JZ UK

**Keywords:** Palliative care, Qualitative research, Older persons, Frailty, Functional decline

## Abstract

**Background:**

The experiences of people with cancer and organ disease have been described across different dimensions of need as they approach death. Such information is lacking for frail older people approaching death, but could highlight how a palliative approach might be relevant for this population.

**Methods:**

Cognitively intact, community dwelling adults considered to be moderately or severely frail were recruited from a medical day hospital. Those recruited nominated an informal carer and case-linked professional. Qualitative in-depth serial interviews with older people and their informal carers were conducted over an 18 month period, and single interviews with case-linked healthcare professionals. Interviews were recorded, transcribed and narrative analytical techniques were used to compile case studies.

**Results:**

Thirty-four participants (13 patients, 13 informal carers and 8 healthcare professionals) completed 40 individual, 14 joint and 8 professional interviews. Five patients died during the study. The analysis highlighted a dynamic balance between losses and adaptations. Three typical patterns of multi-dimensional change emerged. 1) Maintenance of psychological and existential well-being with a gradual social decline mirroring the physical deterioration. 2) a gradual reduction in both psychological and existential well-being. 3) a marked downturn in social, psychological and existential well-being before death. Frail older people sustained their well-being through maintaining a sense-of-self, garnering support from carers and community structures, and focusing on living from day to day. Their well-being lessened when they lost their sense-of-self, feeling alienated from the world, and confused over the cause of their circumstances. Death remained distant and ‘undiagnosed’. Social and community frameworks were essential for supporting their well-being.

**Conclusions:**

Multidimensional end-of-life trajectories for frail older people differed from those with other conditions. Alleviating psychological, social and existential distress should be a priority of care as frail older people reach the end of life. The current palliative care model is problematic for this group. Care should address future concerns and not necessarily involve a focus on death or place of death.

## Background

The World Health Organisation (WHO) has called for a palliative care approach to be enshrined in the care of older persons, that ‘improves the quality of life of patients and their families through the prevention and relief of suffering by means of early identification and assessment and treatment of pain and other problems, physical, psychosocial and spiritual’. Furthermore the WHO in 2014 resolved that a palliative care approach should be integrated in all settings [1]. In the UK, the Scottish government has committed to delivering palliative care on the basis of clinical need rather than diagnosis [2] while the English End-of-Life Care Strategy includes frail older people in its call to develop services tailored to the needs expressed by patients and their carers [3]. Current palliative care services remain predominantly for those with cancer [4].

Three typical end-of-life patterns or trajectories have been described: 1) acute and relatively predictable, typically cancer, 2) gradual decline with intermittent exacerbations, typically organ failure and 3) a gradual more prolonged dwindling, classically frailty or dementia with death occurring after a long period; often years [5]. These are trajectories of physical function yet dying is a four-dimensional activity: physical, psychological, social and spiritual or existential [6]. Exploration of the end-of-life trajectories according to these four dimensions for people with organ disease and cancer do not clearly correspond to physical trajectories but change according to other factors [7]. Such holistic and dynamic information can help to guide palliative care policy and interventions.

Evidence of how social, psychological and existential dimensions of needs of frail older people change over time towards the end of life relates mostly to physical dimensions. [8, 9]. In general, these describe a pattern of a gradual reduction in functional capacity and increasing frailty leading to a more rapid decline towards death. There is, however, a wide degree of variation between individuals even towards death. It is within the non-physical dimensions for the trajectories of defined frail older people that there is a gap in the literature. Research using ‘quality of life’ measures to investigate the experiences of frail older people offers limited detail of how different dimensions interrelate over time. Furthermore, research has tended to focus on frail older people living in care homes, with specific morbidities or with dementia. Research which integrates the different dimensions of need and understands the changes that can occur in psychological, social and existential well-being and how these inter-relate and change over time for frail older people living in the community is needed [10, 11].

Frailty was defined in this study as socially constructed and a dynamic process involving the person and their broad social environment emerging at the tipping point between social and functional dependence and independence [12]. Most evidence of physical, social, psychological and existential dimensions of experience for frail older people relates to physical dimensions. We aimed to understand the experiences, needs and priorities of frail people living at home and their lay carers towards the end of life to inform effective models of supportive and palliative care desirable to frail people.

## Methods

To best meet our aims we selected a qualitative longitudinal (QLL) approach underpinned by a constructivist theoretical perspective, which emphasises that meaning and understanding is achieved socially rather than in isolation from the world around us. Qualitative research offers the ability to investigate complex, contextually based phenomena while QLL offers the potential to gain an understanding of how experiences evolve or change across time. The prospective QLL method was chosen to elicit the changing experiences and care needs of people at the end of life [13]. A multiperspective approach was employed in order to capture the accounts of informal and formal carers, providing a rich understanding of patient experiences [14].

### Recruitment

We recruited participants from a medical day hospital between July 2010 and March 2011. They were identified by clinical staff. Those over the age of 75 with a Mini-Mental State Examination (MMSE) [15] score of above 23 intact were considered. Participants were moderately or severely frail according to the Clinical Frailty Scale (CFS) were invited to take part [16]. Sampling was purposive and aimed to approximate the gender balance, co-habitation status, and demographics of those over the age of 75 (ONS 2006). Participants were invited to nominate an informal carer and the health or social care professional who was most involved in their care.

Written consent was obtained from each person involved, and all were reminded that they could terminate the interview at any time. If a participant withdrew or died without having taken part in a follow up interview a further participant was recruited. Verbal consent was ensured at subsequent contacts.

### Data generation

An experienced qualitative researcher (AL) trained in psychology and nursing conducted the interviews up to three times over a mean period of 18 months. Interviews were carried out either individually or jointly with the informal carer according to the needs and wishes of the older person and lasted between 35 and 120 min. It was planned to undertake interviews every 6 months, and participants were telephoned every eight weeks in order to evaluate on-going circumstances in an attempt to capture change. Participants made the decision whether they wished to undertake a follow up interview or to delay this for another eight weeks. Single case-linked professional interviews were carried out to contextualise the older person and carer interviews. These were undertaken by telephone or face to face according to the preference of the professional and lasted between 10 and 55 min. All interviews were audio-recorded and transcribed verbatim together with detailed field notes.

Interviews utilised a narrative approach and employed open questions to allow participants to fully respond before any prompting, asking for elaboration or opening up further areas for discussions. Interview guides were used flexibly to maintain the person centred focus. Questions aimed to cover the holistic model of the illness experience broadly incorporating dimensions of physical, social, psychological and existential experience. Examples were questions about mobility or physical symptoms and about social relationships with friends, family and communities. Also elicited were the older person’s thoughts about the future. Further questions covered communication with professionals and health and social care services. Follow up interviews explored issues that had emerged in the earlier interview, to investigate change and to broach topics that had not featured highly or been avoided previously. Professional interviews sought to elicit their understanding of the older person’s current and potential future situation and needs and perceived challenges in providing effective care. Bereavement interviews were carried out to gain an overview of the circumstances and story of the older person’s death.

### Data analysis

AL analysed all interviews using a dialogic/performance method that ensured close attention to the role of the researcher and the social circumstances and setting in how the narrative was produced and analysed. The Voice Centred Relational Method (VCRM) [17] was chosen because it requires close attention to the voice of the participants in the different relational contexts of their lives [18]. Four distinct readings of the transcript are advocated in this method where the investigator listens initially to the overall story then to the participant’s voice in its relational context i.e. in relation to the self, to social relationships and in relation to the wider social, cultural and historical context. A key advantage of using the VCRM is that it promotes a depth of involvement with the data. It forces the analyst to think across the key areas of the overall story and really hear the voice of the individual in their relationships. The method also includes a built in reflexive component, where the researcher is required to explore their own reactions and biases to the interview. The different dimensions of experience voiced by the participants were captured, while the possibility of fragmentation of the narratives was minimised.

Professional interviews did not take a narrative course so were analysed ‘in relation’ to the participant’s story looking for themes that concurred with, differed from or clarified the older person’s account. Regular discussions within the multi-disciplinary research group added depth to the data interpretation.

The data were structured as case studies [19] centring on the older person but including the informal and professional carer. This aimed to allow each story to emerge, rather than to look for overarching general themes. Full transcripts and analyses were re-read prior to follow up interviews and analyses. Longitudinal case studies were compiled with the aid of a visual framework that plotted themes and issues according to the VCRM at each time point. The four readings of the VCRM formed rows and each interview time point formed columns. This made it possible to compare directly across time points. Links were then drawn between time points to evaluate change or lack of change, to appreciate how specific issues became more or less prevalent or otherwise changed over time. Individual case stories were then written with change across the time points in physical, psychological, social and existential well-being illustrated graphically for cross case comparison.

### Quality assurance

Decisions taken throughout the stages of recruitment and data generation were recorded in field notes. The analytic decision trail was evident via the proscribed procedural stages of the VCRM and documented within the longitudinal frameworks. This study is reported in line with COREQ guidelines for qualitative research [20].

## Results

### Overview of findings

We recruited 13 patients, 13 informal carers and eight case-linked professionals. Two participants declined to take part and none that were recruited chose to drop out of the study. The average age of participants was 86 (range 76–92) years of whom eight were female. Nine participants were widowed, three married and one single. Seven participants lived alone, two lived with an adult child, three with a spouse and one with a sibling. Five participants died during the study (See Table 1). Eight professionals comprising two agency care workers, five general practitioners and one occupational therapy assistant were interviewed. A total of 54 patient and carer interviews were carried out, of which 14 were joint, 37 separate and 3 were bereavement interviews (See Table 2). One older person interview was conducted at the outpatient unit, one was carried out in an inpatient unit and all others were at the older person’s home. Two carer interviews were carried out by telephone and the others in either their or the older person’s home. Participants and carers were allocated identifying numbers that were used to ensure anonymity. Pseudonyms were then allocated following analysis.

**Table 1:** Participant details: pseudonym, living status, marital status. Ages ranged from 76 to 91 years

Participant pseudonym	Living Status	Marital Status	Survival Status
Mrs A	Offspring	Widowed	Survived
Mr G	Spouse	Married	Survived
Mrs W	Alone	Widowed	Survived
Miss P	Alone	Unmarried	Survived
Mrs B	Spouse	Married	Died
Mrs R	Sibling	Widowed	Survived
Mrs H	Alone	Widowed	Died
Mrs O	Alone	Widowed	Survived
Mr M	Alone	Widowed	Survived
Mrs K	Spouse	Married	Died
Mr C	Offspring	Widowed	Survived
Mr I	Alone	Widowed	Died
Mrs E	Alone	Divorced	Died

**Table 2:** Number and length of participant interviews (8 professional interviews were also carried out)

	Time point 1	Time point 2	Time point 3	Total
Single	16	12	12	40
Interview Length	25–95 mins	30–95 mins	30–85 mins	
Joint	5	6	3	14
Interview Length	30–55 mins	35–70 mins	30–35 mins	
Total	21	18	15	54

Three different patterns of multidimensional needs were identified. Each is described by its pattern of change, graphically plotted to visually represent the changing multidimensional needs of the older people.

### Pattern 1: The coping narrative - managing to balance loss with adaptation

**Fig. 1:**
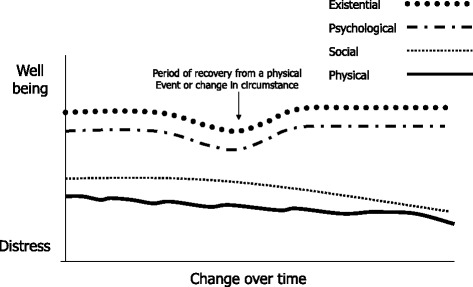
Visualising the ‘coping’ narrative. A qualitative illustration of how the different dimensions changed in relation to each other

Overall this group described gradual physical deterioration with small dips that mirrored social decline (see Fig. 1). Existential and psychological well-being was maintained as effective adjustments were made to increasing losses and physical and social changes. The participants sought to adapt to losses by ‘striving for a new normal’, managed to maintain social connections and focused on living from day to day as opposed to thinking about the future. There were 5 older people in this group. They were followed for 12 to 19 months with the exception of one participant who died 2 months after the first interview. Interviews were carried out at an average of 8 month intervals.

### Striving for a new normal

The older people had experienced many life changes and losses but sought ways to adjust to their circumstances and integrate them into a ‘new normal’. For example in the early interviews Miss P expressed her desires for restitution of her previous function while Mrs A lamented her losses.

*”Once I get on my feet and into that taxi again, I’ll be up [to the city department store] like a shot - If I could just get back [to where I was]”* (Miss P, TP1)

*“but I can’t do anything like the cooking I used to do and the baking, no, I can’t. So, so much of my life’s changed”* (Mrs A, TP2)

However by the third interview both Mrs A and Miss P appeared more resigned to the permanence of their new situation.

*“Well, I might get a bit better but, you know, every time I go in [to hospital] and something happens you never quite get back to where you were”* (Miss P, TP3)

*“I’m getting more used to the fact that I’m not out now, I just say “well, it’s another day you won’t be out” and just sort of say “what will I do today”* (Mrs A, TP3)

*“Well I think I am so lucky because [the fall] could have happened to me years ago and I would still have been damaged (mm hm) I suppose I would have been able to do more because I would be younger (mm hm) because my age has a lot to do with it too”* (Mrs A, Time point (TP 3)

Participants in this category had retained a sense of self that was linked and integrated with the self of the past.

*“Well I’ve always been a person with a deep attachment to the family and really since my husband’s illness my life really revolves around him and I think that’s what keeps me going”* (Mrs B, TP1)

Some of the people interviewed managed this link to their sense of self by finding ways to continue lifelong passions as illustrated by Mrs A

*“Oh yes I miss the tennis terribly….oh it was my life,” “this week will be very hard for me because it’s Wimbledon and I have to watch it and never be able to go again and never see them”* (Mrs A, TP1)

By the third interview Mrs A had begun to schedule her life around televised tennis tournaments.

*“Well, if it wasn’t for reading I don’t know what I would do – and television but I’m not all that keen [on television programmes], I mean, I like things like tennis on the television”* (Mrs A, TP3)

### Maintaining social connections

Older people in this group retained a sense of connection to the world through social and community support networks.

*“And [we enjoy the church club] and the people are collected and brought to it, it’s just a man speaking or sometimes we have somebody showing slides or we somebody speaking on a very interesting subject………………..and all the old people are collected and taken to it”* (Mrs R, TP1)

*“Oh, well… I’m a sort of social buddy, you know, I’ve always been one of a big family and……….you know, when I go with the girls in the bus they all said “oh hello, come on!” you know, the things like that and like we have a good laugh”* (Miss P, TP3)

Further social connections were maintained through the support of their informal carers and carers when they had an established relationship with them.

*“And I can go back with her lot and with the other side [of the family] away back 200 years and name them all sort of thing and the ages and the dates and the stuff, and so she has and she’ll say “of course, great grandmother so and so” and she likes nothing better, when I go there she’ll get back into these subject, that is [she] really loves to do that”* (Niece of Miss P, TP2)

*“They’re interested in the carers that come in and they want to know about them and they want to have this therapeutic relationship essentially”* (Daughter of Mrs R, TP1)

They also tried to balance the situation by reciprocating care where they could.

*‘I give a lot away to charity, I’m going to give £100 to the church. Because the roof’s falling to bits.’ (Mrs R TP1)*

### Focusing on living from day to day rather than looking to the future

The future was not generally contemplated. Although death was acknowledged it remained distanced and in the abstract.

*“Because I mean although I say I’m nearly 90, you’ve got to expect these sort of things, I’m quite willing to live a long time if I can”* (Miss P, TP3)

Instead the older people focused on living day to day.

*“I really just take life one day at a time”* (Mrs B)

Some of these older people had begun to weigh up the costs versus benefits of medical investigations and treatments to their own experience and future wishes and with the support of a well-connected GP were able to transition to an implicit palliative model of care, eschewing what they considered may be intolerable interventions.

*“They were thinking of doing some investigations about her bowel problem but they said “just leave it alone”* (Carer of Mrs A, TP3)

*“And are you quite happy [about that]?”* (Interviewer)

*“Yes”* (Mrs A)

*“I mean she got out the hospital and she just…..they wanted to do [the endoscopy to investigate anaemia] again, and she just said no, there was no way and the [GP] came in the next day and he says “no, no”* (Daughter of Mrs W, TP3)

These older people simply wished to carry on life as it was.

*“As long as I’m able to be in this house, I’ll be quite happy”* (Mrs W, TP1)

However there were situations that they wished to distance themselves from specifically developing dementia, or moving to a nursing home.

*“Whereas if you had no mind and a good body what would you do, you’d just go shuffling around wondering where you were, mind you you’d be quite happy doing that, but I wouldn’t be happy doing that I would rather be away than have that”* (Miss P, TP3)

*“I’ve done a lot of [visiting people in a nursing home]”* (Daughter of Mrs A, TP3)

*“And what are your thoughts on…?”* (Interviewer)

*“Well, I keep away”* (Mrs A)

Figure 1 graphically illustrates changes across the different dimensions of need within the ‘coping’ narrative. This visually represents potential change rather than a quantifiable reality or prediction. Overall these older people described gradual physical deterioration with small dips that mirrored social decline. Existential and psychological well-being was maintained as adjustments were made to increasing losses and physical and social changes.

### Pattern 2: The struggling narrative - struggling to balance loss with adaptation

With this background of gradual physical decline and gradual reduction in social connections, psychological and existential well-being began to dip. Fears of the future began to build. Existential and psychosocial downturns occurred following illness or changes in circumstances and the older person did not fully adapt to a new normal. Similar to the coping narrative these were also life stories but current difficulties were more prominent. Such participants struggled to balance losses with adaptation which led to a loosening grip on the sense of self. They began searching for a cause for their difficulties, expressing a sense of loneliness and futility and building fears of the future. Death also began to enter the accounts but remained distant and abstract. There were 5 older people in this group. They were followed for 12 to 23 months. Interviews were carried out at an average of 9.5 month intervals.

### Searching for a cause

These older people struggled to make sense of their circumstances as old age alone did not offer an adequate explanation. Starting points or contributing factors could be pointed to and old age was acknowledged, however a clear understanding or cause for their circumstances was harder to find and they began to question why they were struggling as they were.

*Mrs K Is going to be seen [at the hospital] then with a view to saying “look please tell us what’s going on, what’s the prognosis?” because we’ve failed to get a proper diagnosis”* (Husband of Mrs K, TP1)

*“I’m just curious as to why your body….and apparently it looks alright, as it were, and yet it doesn’t work?”* (Mr M, TP3)

Although Mr M had accepted that he would not regain his mobility he began to really question why this was given that there was no clear medical illness to attribute it to.

These frail older people were striving to return to a level of previous function and, in the absence of adapting to a new normal, were struggling to maintain links with their old sense of self. ‘How they were’ did not fit with their sense of ‘who they were’. Stories of previous selves featured heavily in these accounts.

*“I can’t believe he’s like that, a fitter man you couldn’t find, I mean even when he retired”* (Wife of Mr G, TP1)

*“I was telling [the researcher] that I played football every day”* (Mr G, TP1)

*“‘She’s waiting for the next stage all the time when things are going to get better. I think she’s harping back to the past when she was much more able to manage”* (Carer of Mrs O, TP2)

### Futility and loneliness

Expressions of frustration and sadness became frequent.

*“I’ve been used to working all my life and I’ve never bothered with anybody but I can’t get used to this sitting about* (Mr C, TP2)

*“I mean actually of all my problems frustration is the main one, just not being able to do things”* (Mr M, TP2)

Similarly, there were regular expressions of loneliness.

*“No I don’t see many [friends] nowadays. I used to go regular to the football, I used to travel all over but I don’t see anybody now”* (Mr C, TP1)

*“But it’s a long day when you don’t see anybody”* (Mrs O, TP2)

Hope was fading for these older people. This was felt especially on discharge from the day hospital that was regarded as a signal that they could no longer be helped although this was not distinct to this group.

*“Oh they’ve given up on me”* (Mrs A, TP2)

*“I’ve been written off”* (Mrs K, TP2)

*“I’ve been flung out”* (Mr C, TP3)

### Building fears

While these participants spoke of future fears, like those in the previous group, these were more dominant and more strongly voiced. Fears were of moving to a care home, developing dementia or becoming a burden.

### Moving to a care home

*“Well we’d have to say the preconceived ideas we’ve got about nursing homes, it frightens the life out of us. I’ve just got a horror of it I’ve got a real horror of it, both of us”* (Wife of Mr G, TP1)

*“We’ll do anything short of ‘A’ bankruptcy or ‘B’ going into a home”* (Husband of Mrs K, TP1)

*“If I was…if the day came that I wasn’t able to look after myself but I was still compos mentis then I suppose I might have to do something like [move to a nursing home] but I don’t think I’d take kindly to it………I think…….I would just give up the struggle, quite honestly”* (Mr M, TP3)

### Developing dementia

*“What you think is [seeing people with dementia in hospital] is this a view into the future and what’s going to happen to me and that’s what has upset me more than anything and you know am I going to end up like that?”* (Wife of Mr G, TP1)

*“Just so long as I don’t go gaga”* (Mr M, TP2)

### Becoming a burden

*“Mm. And you say about not letting your family down and keeping everything up for them. Can you tell me a wee bit more about what you mean by that?”* (Interviewer)

*“Well, I don’t want to be a burden. They do a lot for me but, erm… [pause] I mean, the idea perhaps of going to stay because I wasn’t able to look after myself and in effect them having to nurse me, I wouldn’t want that” (Mr M TP2)*

*“And I mean they must have their own activities to get on with and we can’t be a burden on them and that’s the LAST thing we’d want to be is a burden”* (Husband of Mrs K, TP2)

*“We try not to be a nuisance”* (Mrs K)

*“Well, who are you being a nuisance to?”* (Interviewer)

*“Everybody else! (laughs) Family and friends. Everybody”* (Mrs K)

These fears were spoken about using personal pronouns of ‘I’ and of a collective ‘you’. The identity of ‘old people’ was distanced and othered as ‘a lot of old folk’ and ‘them’.

*“I’m not sitting with a lot of old folk with their heads on their chest you know”* (Mrs O, TP1)

*“[One man in a nursing home] couldn’t stand it any longer I don’t think, stopped eating, turned his face to the wall and went, not that I was visiting him or close to him but I know of him well and that’s the sort of thing you dread, most people do”* (Husband of Mrs K, TP2)

*“I mean, I still… as I say, it sounds ridiculous but like the [name of day centre], I feel so many of these folks are old folks, they’re old folks mentally and I don’t feel old mentally”* (Mr M, TP2)

The older people experienced feeling conspicuous or shame in their physical situation. This was evident in Mr M’s preference for going to public places where there were many other older people.

*“I think that’s [the garden centre] stress-free because there’s old people there and, you know, he probably doesn’t feel conspicuous”* (Daughter of Mr M, TP3)

Frequently hospital admissions heralded a new degree of uncertainty. However discharge allowed some degree of optimism initially as the older person strove for a return to ‘normal’. It was where this normality was not achieved that a period of adaptation took place. Either the older person re-evaluated their situation to work out a new ‘normal’ or they began to mourn for their lost capacity and fear for the future.

*“Well, I mean, I don’t feel ill in the sense of ……in that sense, it’s just this sort of tiredness and futility, you know, if I drop a thing it’s an effort to get down to pick it up kind of thing”* (Mr M, TP2)

Fears were so diverting, being so frequently voiced along with descriptions of current struggles, as to inhibit the adaptive strategy evident in the coping group of how to adapt to a new normal and focusing on living from day to day.

### Death enters the accounts but remains distant

Death was spoken about, at times, yet remained abstract and did not take on the appearance of being expected.

*“Well we’re both in our 80’s, well I’m 95 and Mrs K’s 84, we don’t really have statistically great expectations of life you see. Sorry but you have to face it. If it is Parkinson’s disease there is no cure, there’s palliative treatment and it can be good for another four or five years at least”* (Husband of Mrs K, TP1)

When Mrs K died after admission to hospital with a heart attack her husband described his despair at having failed to find a cause for her difficulties.

*“I was looking perhaps stupidly, perhaps stupidly I was looking for a solution, if not a cure. We never found a solution”* (Husband of Mrs K, TP3)

He felt that he should have saved her.

*“Well I didn’t save her did I”* (Husband of Mrs K, TP3)

*“Do you mean her life…at the end?”* (Interviewer)

*“Yeah…I’ve said it again. I’ll say it once more; grief can be a selfish thing but there’s no selfishness about the efforts, I was totally dedicated to helping”* (Husband of Mrs K)

**Fig. 2:**
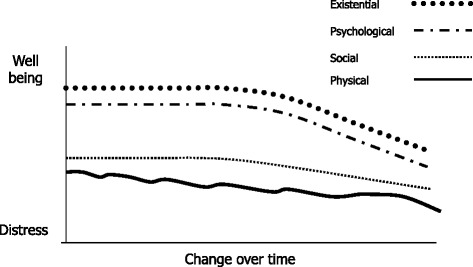
Visualising the ‘struggling’ narrative. A qualitative illustration of how the different dimensions changed in relation to each other

Figure 2 graphically illustrates changes across the different dimensions of need within the ‘struggling’ narrative. This visually represents potential change rather than a quantifiable reality or prediction. Overall adjustments to physical and social losses become harder to maintain. Psychological and existential well-being began to dip. Fears of the future began to build and social connections to loosen.

### Pattern 3: The overwhelmed narrative - losses overwhelm adaptation

Broadly these stories told of a gradual physical decline that steepened towards the end. Social, psychological and existential well-being declined sharply with death occurring shortly afterwards. While some life story was included the focus appeared to be on daily struggles. For these older people, age alone was not enough to account for their circumstances which no longer made sense having not recovered as they hoped. Participants told of losses that overwhelmed their capacity to adapt. Their accounts told of increasing physical difficulties and increasing social isolation. They began losing the capacity to adjust, struggled to hold on to an important and deeply valued issue and finally their future fears became reality. There were 3 older people in this group. They were followed for 13.5 to 19 months. Interviews were carried out at an average of 7.5 month intervals.

### Increasing physical difficulties

Physical function declined with symptoms such as increasing pain and immobility. For Mr I, severe pains in his feet caused him a great distress. His GP had been unwilling to try stronger medication as she felt it would dangerously effect his balance. The GP had considered hospital or nursing home admission for a trial of medication but along with Mr I’s daughter thought he would resist this. Yet when this was explicitly put to him his distress was such that he claimed he would have done anything.

*“How would you feel if the doctor said that you could try strong painkillers but you might have to be in hospital to see that it’s okay and doesn’t make you unsteady? How would you feel about that?”* (Interviewer)

*“I don’t mind if they take away that pain. The pain, just the pain in my legs and that, I don’t mind”* (Mr I, TP3)

### Increasing social isolation

The older people became increasingly isolated socially and expressed a sense of disconnection from the outside world.

*“I just want to be normal and to see people and have them visit me and to be able to go out in my wheelchair once in a while and talk to people”* (Mrs E, TP2)

*“We’re the forgotten army”* (Mrs H, TP2)

*“That’s what’s wrong to me, no matter where I’ve been, now I’ve got to this age all my friends are away, there’s none left”* (Mr I, TP2)

*“People are very patient when they come and see you but they’re in with another one. That’s you, you’re going over a wall or somewhere and they’re on the other side”* (Mr I, TP3)

### Losing the capacity to adjust

Life no longer made sense in terms of how they had lived in the past. The connection between the self of the past and the self of now became broken. These older people strove to understand why they were the way they were. The absence of a clear cause for their circumstances inhibited the capacity to adjust and to reconstruct a new normal.

*“I don’t know, just deeper things, you know, he was on yesterday about the doctor, he hasn’t… he said “what’s actually wrong with me?”, I said “it’s old age” I said “and your heart’s not working properly” – “no, it’s not that” he said “it’s something, it’s sclerosis or something, it’s… I think multiple sclerosis, I’m not sure” and then he said “it’s the worst one possible that you could have and I know that, that that’s what it is but she’s not telling me that, you’re not telling me that” and I said “because that’s not the case”* (Daughter of Mr I, TP3)

### Struggling to hold on to an important issue

Participants each repeatedly emphasised a single issue that seemed to be of deep importance that they insisted they would not relinquish.

*“I will not go into a nursing home, I won’t do it”* (Mrs H, TP1)

*“I just want to live out my days in my own home”* (Mrs E, TP1)

*“I’ve had to do that all my life, keep going. Discipline that’s the centre of everything, discipline. You discipline yourself to do something every day and you do it, no messing about. If you start to slip you remember ‘discipline….you’ve lost the place’ I’m a disciplined person”* (Mr I, TP1)

These factors signified a final point which could give life meaning and purpose and allow them to still live their lives in a way that reflected who they felt they were. They also offered the sense of retaining a degree of self-determination in the face of ever reducing autonomy.

When unable to control their world via their physical capacity, these people emphasised the power of the voice, their choices were enabled through their informal carer. As their informal carers increasingly struggled and could not continue to act as the facilitators for their choices the older people suffered an acute loss of self-determination.

As Mr I’s narrative exemplifies he strongly strong resisted any outside help at the first interview, justified by his lifelong belief in being stoical and autonomous.

*“Aye, I told them, I says “one of these days” I says “I might need you but not just now. That’s right, I’ve had to be disciplined all the time, and discipline other people”* (Mr I, TP1)

*“The old soldier”* (Daughter of Mr I)

*“It’s as simple as that”* (Mr I)

However, with ever increasing falls his daughter was unable to offer the level of support that he now required and so he conceded to using a personal alarm and having help from social carers. He later described himself as useless.

*“I can’t do anything myself. Useless”* (Mr I, TP2)

His daughter described this as an anathema to him.

*“I think he sees [having outside help] maybe as giving in”* (Daughter of Mr I)

His loss of freedom was very distressing.

*“And you see, I’m in a cage and I can’t get out”* (Mr I, TP2)

What followed was a degree of despair and anger.

*“You feel you want to walk out there and walk under a bus”* (Mr I, TP3)

*“Oh dear”* (Interviewer)

*“You just get so……I get so frustrated and angry”* (Mr I)

Similarly Mrs E voiced resistance, supported by her daughter that she would remain in her own home where she felt she could maintain her sense of autonomy.

*“My daughters fought to get me home; the doctor there said no, I had to go into a nursing home or a hospital where they looked after me all the time. I’m not an inmate”* (Mrs E, TP1)

This later changed when her daughter could no longer manage the care Mrs E required to lamenting her situation as alien to who she was.

*“No, I think it’s a lot to do with the fact that I’m in and out of hospital and the fact that I don’t seem to have the control I had; I’m one of these people that all my life I’ve wanted to be very much in control and…right now I feel though that everything’s just getting away from me”* (Mrs E, TP2)

Mrs E was also deeply distressed that a young carer that had visited her regularly and with whom she had a close and supportive bond with had been removed from her care without explanation.

*“But she was young and had a young slant, not er… It was a case of “well, you’ve reached an age where things will not happen now, you’ll have to just take things as they come and take it easy” – I wasn’t ready for that. That sounds selfish, I know, but I just wasn’t ready for that kind of life and I still don’t feel ready for it. I feel I’d like to see people, I’d like to know what was going on, I’d like to know what’s happening about politics and government, all sorts of things, whereas at my age I should be thinking about other things but no, I’m still that way inclined, that I’m very much interested in what’s going on in life”* (Mrs E, TP2)

Like Mr I, Mrs E now despaired and lost interest in her previous activities.

*“And usually I have newspapers but I don’t know what’s happened to them. And I seem to have lost interest in reading…which is not like me I’m an avid reader, always have been………I just have no interest in anything at the moment. The days just come and they go and I don’t feel it’s getting any better”* (Mrs E, TP2)

Mrs E longed for her life as it was and was profoundly distressed at a psychological and existential level. She had previously expressed what made her life worth living.

*“As long as I’ve got the health and strength to enjoy my television enjoy my books and listen to debates that I can still understand and enjoy and as long as I see my family that’s me, I’m contented with that”* (Mrs E, TP1)

Yet at the later interview her personal autonomy so clearly offered her meaning and purpose in life and made it worth living that she despaired at its loss.

*“I think I’ve got this idea that if I was at home things mightn’t always be smooth and that but at least I’d be in my own home and I’d be in control of things, whereas now I just feel I’m an onlooker”* (Mrs E, TP2)

### Future fears become reality

Left with no choice but to accept what they had struggled to resist future fears became realities and resisted identities became the new self-identity.

*“I didn’t think I’d live to this age to finish up like this”* (Mrs H, TP2)

*“This is not a life, just an existence”* (Mrs E, TP2)

Death moved to the foreground of the story and was acknowledged and accepted.

*“You say to yourself well is it worth it, all this nonsense?”* (Mr I, TP2)

*“I want to go home. And I don’t want to be a nuisance, I just want to be there in the background, have my cup of tea and whatever it is that’s going and just let my life drift that way”* (Mrs E, TP2)

**Fig. 3:**
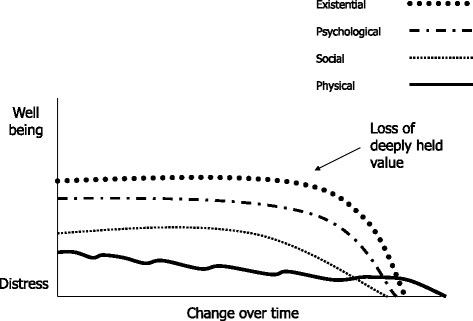
Visualising the ‘overwhelmed’ narrative. A qualitative illustration of how the different dimensions changed in relation to each other

Figure 3 graphically illustrates changes across the different dimensions of need within the ‘overwhelmed’ narrative. This visually represents potential change rather than a quantifiable reality or prediction. General physical decline continued, but without any obvious physical trigger a tipping point was reached when informal carers became unable to continue their support for the older person’s preferred situation which forced the older person to relinquish a deeply held value. When this was lost the older person became increasingly socially adrift, experienced profound psychological distress followed by existential despair.

## Discussion

### Summary

All participants experienced a gradual physical decline and a diminishing social world from already low levels. Psychological and existential well-being dipped in response to changes in circumstances or periods of physical or emotional illness. Well-being related to whether the older person was able to adapt to their new situation. Some reached a tipping point where carers were unable to continue to support the older people to live in the way that reflected their sense of self. They then experienced increasing psychological and existential distress with a social death prior to dying. Frail older people’s sense of meaning and purpose in life remained steady in the coping narrative but eroded gradually and finally in the other scenarios. Factors that supported or challenged the capacity for older people to sustain their well-being are now discussed.

### Factors that supported older people to sustain their well-being

#### Maintaining a sense of self

In response to changing circumstances or physical difficulties older people attempted to adapt to their circumstances by maintaining links with their sense of self. This took a number of forms. Initially they sought to put their illness or incapacity behind them and return to ‘normal’ [21]. When this was not achieved they tried to reconstruct circumstances that related to their sense of self [22, 23]. They found attenuated ways to continue with previous interests and hobbies or embarked on new ones suited to their new circumstances. They compared themselves with others they considered less fortunate, frequently evoking images of those they had seen neglected in hospitals or who had moved to nursing homes. These strategies functioned to distance the reality of their own frailty by placing a worsened state away from the self and onto the distanced ‘other’ and to normalise their own circumstances [24].

Those interviewed gained existential comfort in ways that related to their sense of who they were. A frequently voiced focus on family relationships reinforced links to the previous sense of self and resonated with the experience of older housebound people [25]. Establishing a relationship with a social carer enabled them to convey their values, their story, and, their sense of who they were’. Finally, those interviewed spent time reminiscing, a strategy which enhances psychological well-being [26, 27], enabling a connection to the lifelong sense of self. Choosing to focus on day-to-day living enabled a sense of anchorage in the world [10].

A broader issue, related to maintaining a sense of self, was a spontaneously voiced and universal desire to remain at home. This may foster a feeling of control that strengthens the link to the past, identity and the self [10]. A person’s home holds symbolic value as a place of familiarity, autonomy and connection to the memories of the past [28]. However dying at home may be problematic for older people [28]. For the participants in this study, their home was a place to live rather than a place to die. Perhaps more importantly, having a ‘home’ may have allowed them to feel that they were avoiding their feared fates. Older people did not express fears about death but considered their own mortality in relation to their advanced age. Dying remained distant and abstract with talk focusing on making wills, power of attorney and getting one’s affairs in order [29–31].

#### Gaining carer and community support

Support from family members and community structures were of primary importance. Family carers provided social contact, helped their older relative to maintain other relationships and, as described in other research [11, 24], supported negotiations with the health and social care system. Carers also provided security and protection and held ‘a central role in maintaining agency and a sense of self through the process of change and adaptation’ ([11]: p15,24). To counter feelings of dependence older people employed strategies that enabled balance in relationships [11, 24] by reciprocating care to others in their world perhaps financially or emotionally. Doing so may help to maintain self-esteem as older people consider reciprocating to be a positive trait [32]. Older people also garnered support from their local communities through neighbours and agencies such as churches, and local services such as day centres and bus services, that encouraged feelings of belonging [24].

### Factors that ‘challenged’ the capacity for older people to sustain well-being

#### Losing the sense of self

Older people experienced losses to self-determination relating to hospital stays, enforced isolation due to restricted mobility, and depersonalisation that chipped away at their capacity to live in a way that reflected their life-long values. Most complained of infantilising and depersonalising care that eroded their status as persons [33]. A common objection was of the evening “tuck-in” service arriving in the early evening leaving the older person wide awake and alone for many hours. The nature of intimate physical care was also challenging and was exacerbated by ever changing agency staff or when carers focused solely on physical tasks.

Being isolated in their homes led to feelings of being trapped and imprisoned. This was especially acute in an institution as their physical limitations denied them the freedom to ‘walk out’ and relates to affronts to dignity at feeling in the control of other people [34, 35]. Although participants described some positive experiences of their care in hospital, many experienced or witnessed neglect and maltreatment of older people [36]. This engendered a fear of hospital stays at a stage where people may have lost the capacity to effectively complain. What is reflected here is less a desire for choice but for compassionate care [37]. In response to the ever reducing physical agency of the frail existence [38] the older people held on to the power and influence of their ‘voice’. Although this deteriorated with accumulating losses, a sense of self could be shored up by the support of a carer. At times the carer could no longer meet the expectations of the older person and became overwhelmed by their caregiver burden [39]. Unable to maintain the carer support, the older person could no longer hold on to a core value that allowed them to connect meaningfully with their sense of self. However, proxy decisions made by informal or professional carers can result in bypassing the views and wishes of the older person.

#### Confusion over the cause of their circumstances

A clear issue in this study, when the narratives became more unstable, was the lack of a clear cause. Those who are frail do not have a diagnosis to explain their circumstances, to displace responsibility, to consider their future or to consider death. When faced with physical deterioration a diagnosed illness can offer an explanation but can also allow distance from the self. One can remain ‘me’ with cancer or heart failure and difficulties can be attributed to the illness yet it is the person herself who is frail.

In the absence of a clear diagnosis to explain their deterioration those interviewed in this study sought to make sense of their circumstances. In the coping narrative, usually a period of hospital admissions or a physical event combined with the inevitable decline of aging was sufficient explanation. However, over the struggling and overwhelmed narratives and what Lloyd describes as losses of the ‘habitual body’ [11] these explanations lost their salience and circumstances no longer made sense.

#### Mounting future fears

Frequent fears were about living in the future as opposed to dying. Aspects of a potential future that concerned the frail older people in periods of stability merged into dread as feared outcomes loomed on the horizon. Specifically, these dreads were dementia, becoming a burden, or moving to a nursing home. Fears of dementia or becoming a burden reflect previous research [11, 29, 40] while the thought of a move to a nursing home seemed akin to a terminal diagnosis. What seems important here is the potential loss of identity [40] and a resisted identity of the frail older person [40]. Becoming frail has cultural undertones of personal failure, possibly attributable to the modern day focus on successful aging [41]. Success, defined as being free of disability and disease, and remaining productive and socially engaged [31, 41] needs personal control and individual responsibility [42]. A dichotomy of success and failure is created with personal failure implied for the unsuccessful [31, 43, 44]. The older people may have compared themselves to this dominant cultural framework that promotes such positive images of the third age as normative. Older people lack alternative positive identities to project the future self, or a diagnosed illness to explain physical incapacities. That older participants experienced a degree of shame at their circumstances was often manifest in the vehement ‘othering’ and distancing of the self from those deemed frail [45, 40]. It seemed that the older persons’ circumstances could begin to reflect back a reality of the self that was intolerable to them; that is an image of ‘real old age’ described as a reviled and rejected state of otherness where life may be deemed not worth living [46].

It was within the overwhelmed narrative that dying became a reality. Peace with the fact, however, was only gained very close to death. Clark and Seymour also found a lack of openness about death and dying in older people. They described fears of dying alone, lack of information about end-of-life care, belief that choices were minimal, and a tendency to focus on small, personally important issues [47].

### Strengths and weakness of study methods

This study is novel in its focus on trying to understand how well-being changes over time across different dimensions for community dwelling frail older people. This was facilitated by the serial interviews and interim phone contact that enabled change to be captured in real time. Rigour was achieved by including multiple perspectives to provide detailed contextual accounts and contrasting and complementary viewpoints and through including a range of ages, genders and living circumstances that reflected the demographics of those over the age of 75. Furthermore all the interviews were carried out by a single researcher while the ongoing analysis was discussed regularly with the research team. The study also focuses on frailty as opposed to specific morbidities which may be more practically relevant for older people. A weakness of the method is that it does not account for frail older people who may lack an informal carer or who have dementia. The qualitative design, and small number of participants necessitated by the design impacts detailed extrapolation to different contexts.

### Implications

Improving the end of life for frail older people means recognising the importance of needs beyond the physical, and enabling the maintenance of a sense of self, supporting carers, and involving community networks. Greatest fears should be planned for to reduce distress while promoting a realistic understanding of normative aging and how death occurs at the end of a long life [48]. Such understanding could be promoted through public health campaigns that promote the acceptibility of interdependence rather than just independence [49]. Furthmore there is a need to highlight positive ways of living with frailty,and reduce the current focus on images of youthfull old age with full physical capacity as being within the grasp of everyone.

#### Palliative care for frail older people

Direct application of the cancer based model of palliative care to support frail older people is problematic because of the inability to demarcate a point of physical decline that would indicate death as imminent in frailty [8]. This study suggests points of decline in social, psychological or existential dimensions that could act as triggers for specific support. Health promotion and care should focus on needs beyond the physical as it was social, psychological and existential domains that caused most distress for the older people in this study. Furthermore, the word ‘palliative’ may be counterproductive for older people who are likely to associate the word with dying and with cancer. A palliative care approach is indicated, without necessarily using the term pallaitive care. Those in primary care may be able to identify a ‘tipping point’ when physical problems, psychological or existential distress cannot be adequately managed [50] and palliative care might be formalised. “Anticipatory Care Planning”, currently implemented throughout Scotland [51], planning for the future and addressing greatest fears whatever they are, may be particularly applicable to frail older people.

#### Addressing greatest fears

Through anticipatory care planning older people could be encouraged to consider their greatest fears. These could then be planned for, in order to reduce fear. If older people live in dread of moving to nursing homes then that needs addressed. Enabling nursing homes to become integrated into and involved with their communities and generally improving the care available in nursing homes may reduce the dread that many older people have of nursing homes.

Models of care that foster interdependency in an ethics of care framework as opposed to focusing on independence as an idealised goal have been proposed as a way of addressing older peoples’ fear of burdening others [49]. The ability to reciprocate in some way could be encouraged and facilitated to mitigate against feelings of dependence and maintaining self-worth [11, 32]. There is certainly a societal and cultural need to move away from the potentially damaging focus of an idealised third age. Furthermore depersonalisation of frailty could be encouraged through descriptions of older people as ‘living with’ frailty rather than ‘being’ frail.

#### Supporting carers and community structures

Community structures can foster a continued sense of belonging, mitigate against social alienation and foster a feeling of self-worth. These should be drawn on and encouraged when considering care for frail older people. Relationships with social care staff can be vital in supporting older people, especially where there is continuity of care and adequate time. Interventions to assist family carers through direct support or through processes that enable them to build on their existing social networks should be considered [52].

## Conclusions

This study describes three end-of-life trajectories across different dimensions of need for frail older people. These have similarities but also differences to multi-dimensional trajectories in people dying with organ failure and cancer. Rather than a direct application of the current model of palliative care developed to suit the cancer trajectory, an approach directed to identify the needs that older frail people normally experience is necessary to provide patient-centred care. Anticipatory planning for deteriorating circumstances circumvents the need to discuss dying and could help to alleviate future fears. Existing community and social support networks are vital for supporting frail older people to minimise alienation and maintain self-determination in the face of increasing dependence.

## Abbreviations

CFS: Clinical Frailty Scale
COREQ: Consolidated Criteria for Reporting Qualitative Research
GP: General Practitioner
MMSE: Mini Mental State Examination
QLL: Qualitative Longitudinal Research
TP: Time Point
VCRM: Voice Centred Relational Method
WHO: World Health Organisation

## References

[1] World Health Organisation (2014). Strengthening of palliative care as a component of integrated treatment within the continuum of care.

[2] Scottish Executive (2007). Better Health, Better Care: action plan.

[3] The Gold Standards Framework. Gold Standards Framework Position Paper: For the NHS End of Life Care Programme. UK: Edited by The Gold Standards Framework; 2007.

[4] Audit Scotland (2008). Review of Palliative Care Services in Scotland.

[5] Lunney JR, Lynn J, Foley DJ, Lipson S, Guralnik JM (2003). Patterns of functional decline at the end of life. JAMA.

[6] Davies E, Higginson IJ. Better Palliative Care for Older People. Denmark: World Health Organisation; 2004.

[7] Murray SA, Kendall M, Grant E, Boyd K, Barclay S, Sheikh A (2007). Patterns of social, psychological, and spiritual decline toward the end of life in lung cancer and heart failure. J Pain Symptom Manag.

[8] Covinsky KE, Eng C, Lui LY, Sands LP, Yaffe K (2003). The last 2 years of life: functional trajectories of frail older people. J Am Geriatr Soc.

[9] Izawa S, Enoki H, Hirakawa Y, Iwata M, Hasegawa J, Iguchi A (2010). The longitudinal change in anthropometric measurements and the association with physical function decline in Japanese community-dwelling frail elderly. Br J Nutr.

[10] Nicholson C, Meyer J, Flatley M, Holman C, Lowton K (2012). Living on the margin: understanding the experience of living and dying with frailty in old age. Soc Sci Med.

[11] Lloyd L, Calnan M, Cameron A, Seymour J, Smith R (2014). Identity in the fourth age: perseverance, adaptation and maintaining dignity. Ageing Soc.

[12] Kaufman SR (1994). The social construction of frailty: an anthropological perspective. J Aging Stud.

[13] Murray SA, Sheikh A (2006). Serial interviews for patients with progressive diseases. Lancet.

[14] Kendall M, Murray SA, Carduff E, Worth A, Harris F, Lloyd A, et al. Use of multiperspective qualitative interviews to understand patients’ and carers’ beliefs, experiences, and needs. Br Med J. 2010;340:196–99. 10.1136/bmj.b4122 19828645

[15] Holstein M, and Waymack M. The contributions of philosophy and ethics in the study of age. Enduring questions in gerontology. 2006. pp. 177–201.

[16] Rockwood K, Song X, MacKnight C, Bergman H, Hogan DB, McDowell I (2005). A global clinical measure of fitness and frailty in elderly people. Can Med Assoc J.

[17] Brown LM, Gilligan C (1992). Meeting at the Crossroads: Women’s psychology and girls development.

[18] Mauthner N, Doucet A, Ribbens J, Edwards R (1998). Reflections on a voice centered relational method: analyzing maternal and domestic voices. Feminist Dilemmas in Qualitative Research.

[19] Thomson R (2007). The qualitative longitudinal case history: practical, methodological and ethical reflections. Social Policy Society.

[20] Tong A, Sainsbury P, Craig J (2007). Consolidated criteria for reporting qualitative research (COREQ): a 32-item checklist for interviews and focus groups. Int J Qual Health Care.

[21] Frank AW. The wounded storyteller: Body, illness, and ethics. USA: University of Chicago Press; 2013.

[22] Charmaz K (1983). Loss of self: a fundamental form of suffering in the chronically ill. Sociol Health Illn.

[23] Williams G (1984). The genesis of chronic illness: narrative reconstruction. Sociol Health Illn.

[24] Tanner D (2007). Starting with lives supporting older people’s strategies and ways of coping. J Soc Work.

[25] McKevitt C, Baldock JC, Hadlow J, Moriarty J, Butt J, Walker (2005). Identity, meaning and social support (Chapter 9). Understanding Quality of Life in Old Age.

[26] Coleman PG (2005). Uses of reminiscence: functions and benefits. Aging Ment Health.

[27] Bohlmeijer E, Roemer M, Cuijpers P, Smit F (2007). The effects of reminiscence on psychological well-being in older adults: a meta-analysis. Aging Ment Health.

[28] Gott M, Seymour J, Bellamy G, Clark D, Ahmedzai S (2004). Older people’s views about home as a place of care at the end of life. Palliat Med.

[29] Lloyd-Williams M, Kennedy V, Sixsmith A, Sixsmith J (2007). The end of life: a qualitative study of the perceptions of people over the age of 80 on issues surrounding death and dying. J Pain Symptom Manage.

[30] Carrese JA, Mullaney JL, Faden RR, Finucane TE (2002). Planning for death but not serious future illness: qualitative study of housebound elderly patients. BMJ.

[31] Lamb S (2014). Permanent personhood or meaningful decline? Toward a critical anthropology of successful aging. J Aging Stud.

[32] Townsend J, Godfrey M, Denby T (2006). Heroines, villains and victims: older people’s perceptions of others. Ageing Soc.

[33] Hockey JL, James A (1993). Growing up and growing old: Ageing and dependency in the life course.

[34] Andersson M, Hallberg IR, Edberg AK (2008). Old people receiving municipal care, their experiences of what constitutes a good life in the last phase of life: a qualitative study. Int J Nurs Stud.

[35] Chochinov HM, Hack T, McClement S, Kristjanson L, Harlos M (2002). Dignity in the terminally ill: a developing empirical model. Soc Sci Med.

[36] Koch T, Webb C (1996). The biomedical construction of ageing: implications for nursing care of older people. J Adv Nurs.

[37] Borgstrom E, Walter T (2015). Choice and compassion at the end of life: a critical analysis of recent English policy discourse. Soc Sci Med.

[38] Agich G. Dependence and autonomy in old age: an ethical framework for long-term care. UK: Cambridge University Press; 2003.

[39] Miller B, McFall S (1991). The effect of caregiver’s burden on change in frail older persons’ use of formal helpers. J Health Soc Behav.

[40] Lee VS, Simpson J, Froggatt K (2013). A narrative exploration of older people’s transitions into residential care. Aging Ment Health.

[41] Rowe JW, Kahn RL (1997). Successful aging. The Gerontologist.

[42] Lloyd L, Tanner D, Milne A, Ray M, Richards S, Sullivan MP (2014). Look after yourself: active ageing, individual responsibility and the decline of social work with older people in the UK. Eur J Soc Work.

[43] Holstein MB, Minkler M (2003). Self, society, and the new gerontology. The Gerontologist.

[44] Rozanova J (2010). Discourse of successful aging in The Globe & Mail: insights from critical gerontology. J Aging Stud.

[45] Minichiello V, Browne J, Kendig H (2000). Perceptions and consequences of ageism: views of older people. Ageing Soc.

[46] Higgs P, Gilleard C (2014). Frailty, abjection and the othering of the fourth age. Health Sociol Rev.

[47] Clarke A, Seymour J (2010). “At the Foot of a Very Long Ladder”: discussing the end of life with older people and informal caregivers. J Pain Symptom Manag.

[48] McCue JD (1995). The naturalness of dying. JAMA.

[49] Lloyd L (2004). Mortality and morality: ageing and the ethics of care. Ageing Soc.

[50] Jerant AF, Azari RS, Nesbitt TS, Meyers FJ (2004). The TLC model of palliative care in the elderly: preliminary application in the assisted living setting. Ann Fam Med.

[51] Baker A, Leak P, Ritchie LD, Lee AJ, Fielding S (2012). Anticipatory care planning and integration: a primary care pilot study aimed at reducing unplanned hospitalisation. Br J Gen Pract.

[52] Kellehear A, OConnor D (2008). Health-promoting palliative care: a practice example. Critical Public Health.

